# The Microwave Temperature and Humidity Profiler: Description and Preliminary Results

**DOI:** 10.3390/s23208554

**Published:** 2023-10-18

**Authors:** Joan Francesc Munoz-Martin, Xavier Bosch-Lluis, Omkar Pradhan, Shannon T. Brown, Pekka P. Kangaslahti, Alan B. Tanner, Mehmet Ogut, Sidharth Misra, Boon H. Lim

**Affiliations:** 1 Signal Processing and Networks Group, Jet Propulsion Laboratory, California Institute of Technology, Pasadena, CA 91011, USA; javier.bosch-lluis@jpl.nasa.gov; 2 Microwave Systems Technology Group, Jet Propulsion Laboratory, California Institute of Technology, Pasadena, CA 91011, USA; omkar.pradhan@jpl.nasa.gov (O.P.); pekka.p.kangaslahti@jpl.nasa.gov (P.P.K.); 3 Microwave Instrument Science Group, Jet Propulsion Laboratory, California Institute of Technology, Pasadena, CA 91011, USA; shannon.t.brown@jpl.nasa.gov (S.T.B.); alan.b.tanner@jpl.nasa.gov (A.B.T.); mehmet.ogut@jpl.nasa.gov (M.O.); sidharth.misra@jpl.nasa.gov (S.M.); blim@blueorigin.com (B.H.L.); 4 Blue Origin, Kent, WA 98032, USA

**Keywords:** atmosphere, sounding, mmWave, radiometry, calibration

## Abstract

This manuscript presents the Microwave Temperature and Humidity Profiler (MTHP), a dual-band spectroradiometer designed for measuring multi-incidence angle temperature and humidity atmospheric profiles from an aircraft platform. The MTHP bands are at 60 GHz for measuring the oxygen complex lines, therefore at this band, MTHP has a hyperspectral radiometer able to provide 2048 channels over an 8 GHz bandwidth, and 183 GHz for measuring water vapor, which only uses four channels since this absorption band’s spectral richness is simpler. The MTHP builds upon the Microwave Temperature Profiler (MTP) with the inclusion of the hyperspectral radiometer. The instrument’s design, components, and calibration methods are discussed in detail, with a focus on the three-point calibration scheme involving internal calibration loads and static air temperature readings. Preliminary results from the Technological Innovation into Iodine and GV aircraft Environmental Research (TI3GER) campaign are presented, showcasing the instrument’s performance during flights across diverse geographical regions. The manuscript presents successful antenna temperature measurements at 60 GHz and 183 GHz. The hyperspectral measurements are compared with a simulated antenna temperature using the Atmospheric Radiative Transfer Simulator (ARTS) showing an agreement better than R^2^ > 0.88 for three of the flights analyzed. Additionally, the manuscript draws attention to potential Radio Frequency Interference (RFI) effects observed during a specific flight, underscoring the instrument’s sensitivity to external interference. This is the first-ever airborne demonstration of a broadband and hyperspectral multi-incidence angle 60 GHz measurement. Future work on the MTHP could result in an improved spatial resolution of the atmospheric temperature vertical profile and, hence, help in estimating the Planetary Boundary Layer (PBL) with better accuracy. The MTHP and its hyperspectral multi-incidence angle at 60 GHz have the potential to be a valuable tool for investigating the PBL’s role in atmospheric dynamics, offering insights into its impact on Earth’s energy, water, and carbon cycles.

## 1. Introduction

The recent National Academies of Sciences, Engineering, and Medicine 2017–2027 Decadal Survey (DS) for Earth Science and Applications from Space (NASEM, 2018) identified the Planetary Boundary Layer (PBL) as a Targeted Observable (TO) [1]. TOs are identified as high-priority observables for which cost-effective spaceborne observing strategies are not yet mature. PBL is the lower part of the troposphere that extends from the Earth’s surface up to the capping inversion layer or temperature inversion layer, typically located at a height of 1–2 km [2]. This region defines the boundary between the surface-influenced atmosphere and the free atmosphere, thereby mediating fluxes of energy, water, carbon, and other constituents within the Earth system. Given the interdisciplinary nature of PBL science, it is not surprising that interest in PBL arose out of multiple DS panels including Weather and Air Quality, Climate, and Hydrology. The key PBL observables were identified as vertical profiles of temperature and moisture (water vapor), along with PBL height [3]. Four PBL science themes have been identified: Convective PBL, cloudy PBL, PBL and surface interaction, and PBL mixing and air quality [3]. To address these science themes, a mix of observation architectures is required, including ground-based, suborbital, and orbital components. To date, four different types of instruments can be used to directly or indirectly study the PBL: Differential Absorption Lidar (DIAL) [4], Differential Absorption Radar (DAR) [5,6], hyperspectral infrared imagers [7], and hyperspectral microwave receivers. The first two types of instruments, DIAL and DAR, are active instruments, and they require sending high-energy pulses to receive their echo. Instruments that rely on high-power components tend to require large vehicles, limiting their suitability for scalable small satellite constellations. On the other hand, hyperspectral infrared is a technique proven for ground-based instruments [7], but not for top-of-the-atmosphere measurements, which prevents the technique from being implemented globally. Thermal infrared spectrometers have been also implemented in space through the Infrared Atmospheric Sounding Interferometer (IASI) [8], showing accuracies of 1 K, and spatial resolutions of 1 km. However, this type of system is limited only to cloud-free regions. A solution for retrievals in cloud-covered regions is the use of microwave receivers. Low-spectral resolution radiometers operating near the 50–60 GHz or 118 GHz oxygen absorption lines have been used to retrieve coarse-resolution vertical temperature profiles at ~1–4 km vertical resolution [9,10,11,12] from the ground, airborne, and spaceborne platforms. Similarly, systems operating near the 183 GHz water vapor absorption line reliably profile air humidity. However, the lack of a finer frequency resolution and multi-angular measurements limits the retrieval of high-resolution vertical profiles, therefore limiting the ability to precisely measure PBL position based on such vertical temperature profiles. In this manuscript, we present the Microwave Temperature and Humidity Profiler (MTHP) [13] developed at the Jet Propulsion Laboratory (JPL) and deployed in the Technological Innovation into Iodine and GV aircraft Environmental Research (TI3GER) campaign [14] led by the University Corporation for Atmospheric Research/National Center for Atmospheric Research (UCAR/NCAR). MTHP is a multi-incidence angle spectroradiometer at 60 GHz and 183 GHz that aims at retrieving vertical temperature and humidity profiles to enable PBL studies from the top of the atmosphere.

## 2. Instrument Description

MTHP is the second version of the Microwave Temperature Profiler [15,16] (MTP). The instrument was conceived in late 2013 to add water vapor information (183 GHz channels) to the MTP [15,16,17]. Both MTP and MTHP are atmospheric spectroradiometers at millimeter wave frequencies and are conceived to be integrated into airborne campaigns. In the case of MTHP, it was deployed in the NCAR-led TI3GER campaign on board a HIAPER Gulfstream-V (GV) airplane. For the 2022 TI3GER campaign, MTHP underwent a major refurbishment, which included an overall update and the substitution of the 60 GHz backend for a hyperspectral detector.

The instrument is divided into two parts. The first part is the MTHP canister, which includes all the necessary components to perform the radiometric measurements. The scanning servo motor and controller are configured to start automatically upon power on of the 28 V line, which is activated with the airplane take off. This entire module is mounted below the airplane wing. The second part is the instrument control, which is a computer together with a power distribution unit that is installed in a rack inside the airplane cabin.

**Figure 1:**
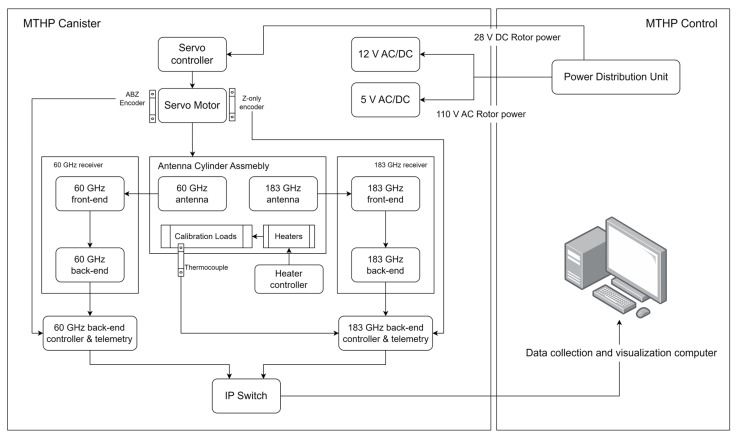
MTHP main block diagram. On the left-hand side of the diagram is the MTHP canister, mounted under the wing of the airplane. On the right-hand side is the control computer that controls and collects telemetry of the 60 GHz and 183 GHz back-end modules, mounted inside the aircraft cabin.

The high-level block diagram of MTHP is shown in Figure 1. As can be seen, both receivers are completely independent to prevent failures in one frequency band from affecting the other. The control and telemetry units are also independent. Both channel command and data handling (CDH) units are Linux-based computers that are connected through an Ethernet (i.e., IP) switch to the main control computer on board the cabin. In the next sub-sections, the instrument design is detailed for the Antenna Cylinder Assembly, the 60 GHz receiver, the 183 GHz receiver, and the control computer.

### 2.1. Antenna Cylinder Assembly Description

**Figure 2:**
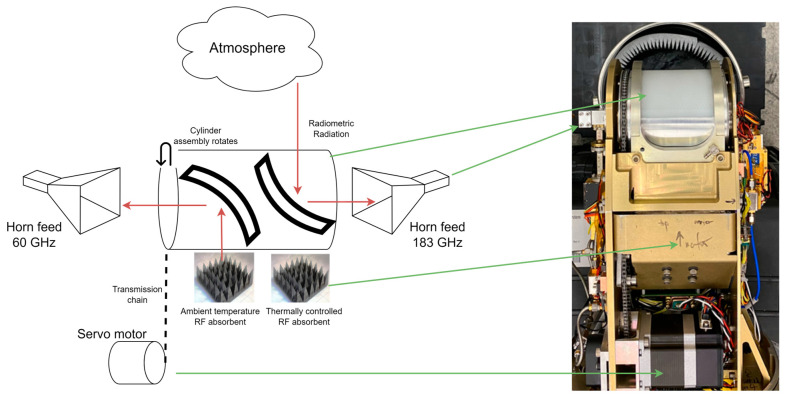
(**Left**) MTHP Antenna and servo assembly block diagram, (**right**) MTHP top view highlighting horn external connection, antenna assembly, and servo motor.

In the same way as MTP [15,17], MTHP performs continuous multi-incidence angle scanning of the atmosphere and its internal calibration targets (i.e., RF absorbent) using a servo motor that is connected to a rotating cylinder (cylinder assembly) that houses the main receiving antenna. The scanning antenna is a paraboloid that directs the signal to a linearly polarized horn antenna. However, as the MTHP is dual-band, two paraboloids and two horns are used, one per band (i.e., 60 GHz and 183 GHz). The rotor moves the paraboloids 360° in the incident angle direction, allowing one to scan the entire atmosphere from nadir to zenith and back. As the antenna rotates, it ‘observes’ two loads located internally. These loads are used for calibration purposes. Figure 2 shows the different blocks composing the cylinder assembly, servo motor, paraboloids, and horn feeds. Note that the two paraboloids are pointing with an offset of 180° with respect to the cylinder assembly revolution axis one from the other, and the feeds are mounted on opposite sides of the instruments, as shown in Figure 2. The rotor performs one rotation every 1.62 s or 225°/s.

The MTHP’s calibration targets are at two different temperatures to provide two calibration points. One of the targets is thermally controlled to a temperature of 333 K, and the second target is left at an ambient temperature and is continuously monitored using thermocouples.

### 2.2. 60 GHz Receiver

The 60 GHz MTHP channel digital high-resolution spectrometer update represents a significant advancement over the earlier MTP version [18]. With the increasing demand for higher resolution mapping of the 60 GHz band for conducting PBL studies, an upgrade was necessary for the receiver back-end. The need to measure a large number of channels around 50–60 GHz was raised to increase the resolution and accuracy of thermal profiles [19].

**Figure 3:**
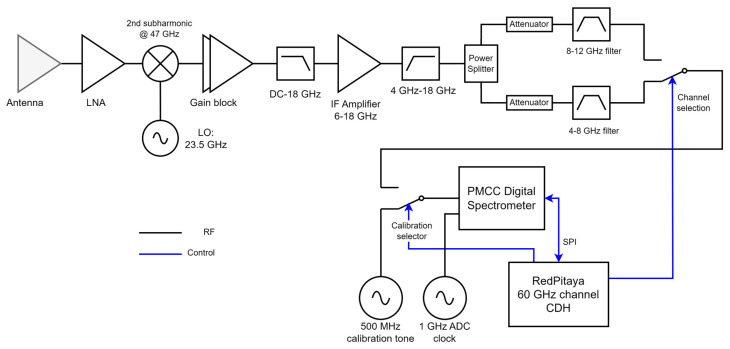
Block Diagram of the MTHP 60 GHz channel receiver.

To meet these requirements, the back-end channel was enhanced with a state-of-the-art Commercial-off-the-Shelf (COTS) high-resolution digital spectrometer. This digital spectrometer, known as the P19800B, is sourced from Pacific Microchip Corporation (PMCC) [20]. The digital spectrometer is capable of providing integrated power spectra of up to 4 GHz of instantaneous bandwidth resolved in 512 to 8192 channels and at user-configurable integration times. The spectrometer is implemented in a low-power application-specific integrated circuit (ASIC) and can be controlled by a Serial Parallel Interface (SPI). Figure 3 provides a detailed block diagram and a general overview of the complete front-end and back-end of the 60 GHz channel receiver. The signal is collected by the antenna and amplified by a first-stage low-noise amplifier (LNA) designed and manufactured at JPL. The signal is down-converted using the 2nd subharmonic of a 23.5 GHz oscillator (i.e., the central frequency is 47 GHz). The upper sideband of the down-conversion is amplified and filtered. The total gain of this receiver chain is ~70 dB.

**Figure 4:**
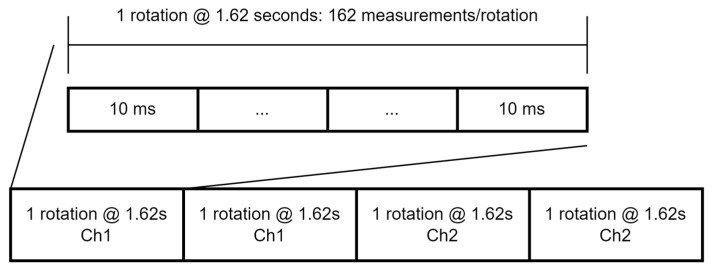
Detailed measurement time-sequence scheme for the MTHP 60 GHz channel.

The MTHP’s goal is to cover as much band as possible in the 60 GHz band. Hence, the entire Federal Communications Commission (FCC) reserved band for Earth Exploration—Satellite (passive) [21] comprised between 51 and 59 GHz is measured. To measure up to an 8 GHz bandwidth using the PMCC chip, a time-division dual channel strategy is followed. This dual-channel strategy is depicted after the amplification blocks in Figure 3. First, the signal is split into two paths using a 3-dB splitter, whereby the lower path is filtered using a 4–8 GHz COTS band-pass filter (RF-Lambda RBPF33G04G08) and the upper path by an 8–12 GHz COTS band-pass filter (RF-Lambda RBPF33G08G12). The output of the filters is directed to a single-pole dual throw (SP2T) switch that switches between the channels. The output of this SP2T is then connected to a second SP2T that is used to switch between the measurement chain (i.e., the previous SP2T) and a calibration signal required for the nominal operation of the PMCC digital spectrometer. The calibration signal is a 500 MHz tone, and it calibrates the gain and offset of the different Analog-to-Digital Converters (ADC) of the PMCC spectrometer. Additionally, a second clock is required for the PMCC ADC to work, selected at 1 GHz. Finally, a Field-Programmable Gate Array (FPGA) board with an embedded dual-core ARM is used to control the PMCC and the calibration/channel selection. The board used is the RedPitaya STEMLab 125, which acts as a Command and Data Handling (CDH) interface with the external system. The digital spectrometer produces a 1024-point power spectral measurement every 10 ms (i.e., 10 ms integration time). Each spectral channel is thus 3.9 MHz wide. Furthermore, the measurement software switches periodically between the upper and lower channels. This strategy virtually produces 2048 3.9 MHz—with points between 51 GHz and 59 GHz. The switch between the upper and lower channels occurs every two rotations. The rotor positions are read by the CDH and synchronized with the channel selector switch. The measurement scheme is detailed in Figure 4. This scheme produces 162 measurements per rotation, with an angular resolution of 2.25° per measurement.

### 2.3. 183 GHz Receiver

**Figure 5:**
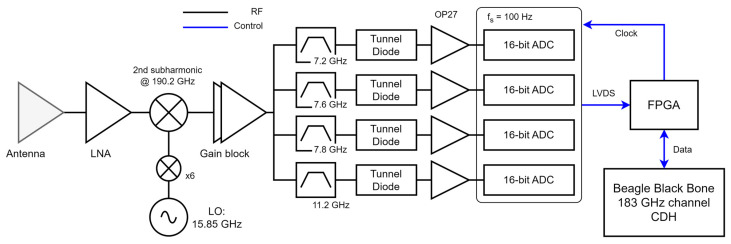
Block diagram of the MTHP 183 GHz channel receiver.

The 183 GHz receiver is used to measure the water vapor absorption band. As compared with the oxygen band, the water vapor band has a continuum spectra with fewer features. For this reason, and to reduce the instrument complexity, it was decided to use a modified version of the MTP receiver backend. The detailed block diagram of the 183 GHz channel is shown in Figure 5. A first-stage LNA is followed by a down-converter that uses the 2nd harmonic of a 95.1 GHz clock (i.e., the central frequency at 190.2 GHz). The lower sideband of the down-converted signal is sampled by the analog backend. Four channels are measured: 179 GHz, 182.6 GHz, 182.8 GHz, and 183 GHz with a bandwidth of 250 MHz each, which are 11.2 GHz, 7.6 GHz, 7.4 GHz, and 7.2 GHz from Figure 5, respectively. The integration time in this case is also 10 ms. Hence, the angular resolution for the water vapor channel is the same as the one in the oxygen channel.

### 2.4. Receivers Characterization: Noise Equivalent Temperature

**Figure 6:**
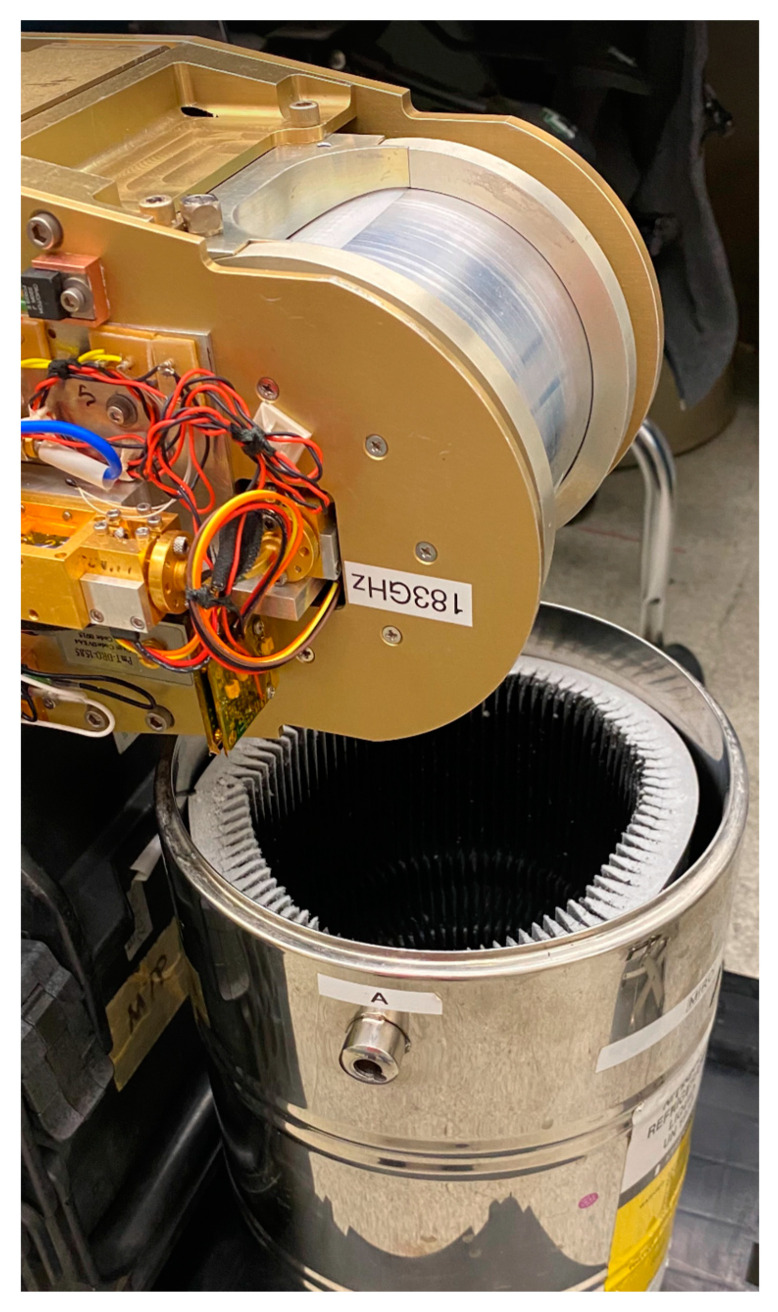
MTHP rotor while on the Y-factor measurement using a liquid nitrogen-filled tank with RF absorbent.

MTHP noise-equivalent temperature measurements were performed on the output of the IF chain (after down-conversion and before digitization) on both 60 GHz and 183 GHz channels. The system noise temperature test is performed using a 77 K liquid nitrogen-filled tank with an RF absorbent (see Figure 6). The antenna is pointed inside the tank and power measurements are performed using a calibrated power meter at each channel. After that, the antenna is pointed to an ambient temperature calibration load at 293 K. The Y-factor is measured, in dB, by subtracting the power value at ambient to the cold target measured power. The results of the receiver’s Y-factor and noise-equivalent temperature (NET) estimates are provided in Table 1.

Given these receiver temperatures, we can estimate the single-channel Noise-Equivalent Temperature Difference (NETD) at 10 ms integration time as ([22], chapter 7):

$$NETD=\frac{T_{Ant}+T_{Recv}}{\sqrt{B_{N_{eq}}\cdot \tau }},$$

where $B_{N_{eq}}$ is the 3-dB noise equivalent bandwidth for a Hanning window of 1.363 times the spectral resolution (i.e., a channel is measured every 3.9 MHz, but the actual noise bandwidth is 5.3 MHz due to the filter roll-off) for the 60 GHz channel, and 250 MHz bins for the 183 MHz channel, with a maximum antenna temperature ($T_{Ant}$) of 300 K (temperature of the thermally controlled calibration load). The NETD assuming no gain fluctuations for MTHP at its native bandwidths and integration time is given in Table 2.

Note that the high NETD for the oxygen channels can be improved using two strategies: Reducing the high spectral resolution (Original $B_{N_{eq}}$ = 5.6 MHz) by averaging adjacent channels and averaging samples with adjacent angular measurement (Original Resolution = 2.5°). This is accomplished in post-processing, giving the end user the flexibility to trade spatial and frequency resolutions for NETD.

## 3. Calibration Strategy

As with other radiometers, MTHP measurements require frequent calibration to compensate for *T_receiver_* variations and gain fluctuations. To calibrate MTHP measurements, a three-point calibration method is used for the 60 GHz channel, and a two-point calibration method is performed for the 183 GHz channel. The two-point calibration method uses the two internal calibration loads (ambient and hot), and the three-point calibration method uses the two loads and the static air temperature (SAT) read by the airplane’s temperature sensor. At cruise altitude, the cross-section of air in front of the instrument is maximum, hence the measured brightness temperature at 60 GHz matches the SAT since the oxygen at the exact absorption band behaves as a blackbody ([22], Chapter 8). Note that the ambient temperature load at high altitudes (altitude > 10 km) is never as cold as the SAT since it is affected by the heat generated by the instrument. This calibration scheme would enhance the estimation of the system noise temperature and gain on the 60 GHz channel.

The first step to calibrate the data is to collocate all measurements with respect to the rotor position. This is performed in post-processing once all data collected by the instrument are retrieved. The rotor position readings are synchronized with the spectral measurement at both 60 GHz and 183 GHz channels to ensure independent data. The rotor data synchronism is achieved by measuring two ABZ optical encoders mounted on the rotor assembly, one for each backend the 60 GHz and the 183 GHz channel.

Bins of 5° are generated between 0° and 360° resulting in a total of 72 angular bins. A drop-in-the-bucket strategy is followed, and each measurement is assigned to its closest angular measure. To obtain a 60 GHz “full spectrum” (i.e., 8 GHz, from 51 to 59 GHz) measurement, four consecutive rotations (6.48 s or 4 rotations) are grouped together and averaged as follows:

- Two consecutive measurements of the lower-side band (51–55 GHz) are averaged together and concatenated to two consecutive measurements of the upper-side band (55–59 GHz), which are also averaged together.
- Four rotations of the 183 GHz channel are averaged together.

Note that since the atmosphere does not drastically change within 6.5 s (assuming travel of approximately 2 km at maximum Airplane speed), averaging 4 full rotations and considering it to be a single sample is acceptable for our case. Therefore, we obtain a multi-incidence angle (5° resolution) acquisition sample from 51 to 59 GHz and the 183 GHz channels together every 6.48 s.

The 72 angular points for each of the 60-GHz 2048 frequency samples and the 4 183-GHz frequency samples are treated as a single atmospheric measurement. Additionally, to reduce the receivers’ noise at the 60 GHz channels, 8 consecutive frequency bins are averaged, producing a total of 256 channels covering 8 GHz of the spectrum ($B_{N_{eq}}=$ 34.6 MHz/channel). This strategy allows us to reduce the NETD of the 60 GHz spectrometer by a factor of 2.48, i.e., from 3.63 K down to 1.46 K.

The calibration continues by taking the 72 angular points for the 60 GHz moderate-resolution spectrogram (31.25 MHz bandwidth, 256 channels) and 72 angular points for 4 measurements (250 MHz bandwidth) at 183 GHz across the entire rotor range of motion every 6.48 s. To calibrate the measurements using the external targets at 60 GHz, points at a rotor angle of θ=[300°,340°, 90°] are retrieved. Those points are facing two internal targets and forward, respectively. For the 60 GHz channel, the airplane SAT and the thermistor measurements of the thermally controlled and ambient targets are compared. The three-point calibration method uses a linear regression to estimate the model: $T_{est}=a+b\cdot m$, with *m* being the raw measurement counts in the spectrogram, and a and b the estimated receiver noise and system gain, respectively. Note that this method differs from other three-point calibration methods as a quadratic function is used to fit the three points. In our case, the resulting regression may not intercept some of the points. Instead, it will look for the most probable gain and offset coefficients based on the three points provided. Further information on radiometric calibration is available at [23,24,25].

**Figure 7:**
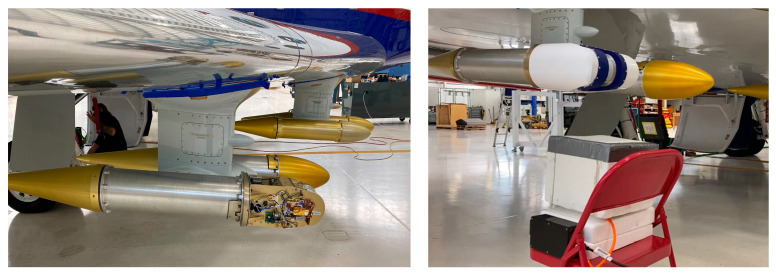
(**Left**) MTHP canister integrated into the GV airplane as part of the TI3GER campaign, and (**right**) external hot load calibration test during integration and validation of the instrument in the GV.

**Figure 8:**
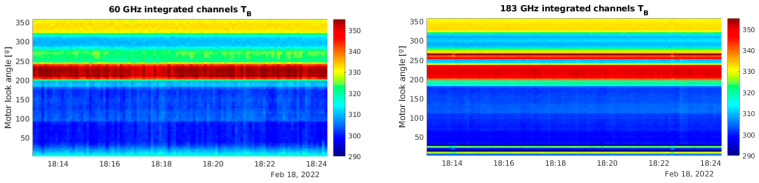
Time series of estimated antenna temperature using external calibration load for several consecutive time-tagged rotations of the averaged spectrum at (**left**) 60 GHz channel and (**right**) 183 GHz channel. Color bar scale is Kelvin. Note that all spectral components have been averaged for visualization. Note that the rotor has an offset and zenith is at 55°.

This calibration methodology was tested on the ground during MTHP integration with the TI3GER GV airplane using an external hot calibration load of 355 K. The measurement setup and its results are shown in Figure 7 and Figure 8, respectively. Note that for Figure 8, the spectral components have been averaged to a single value to ease the visualization. As can be seen in both channels, following the rotor angle, the estimated antenna temperature is 290 K (dark blue) when pointing inside the hangar at room temperature. The external heated load, located at 220°, is at an antenna temperature of 355 K (red). The temperature of the internal ambient load is the light blue line at 300°, and the thermally controlled internal target is the yellow line at 350° (T = 333 K). Note that in this plot, the zenith is ~55°.

## 4. TI3GER Campaign Measurements

### 4.1. Campaign Overview

**Figure 9:**
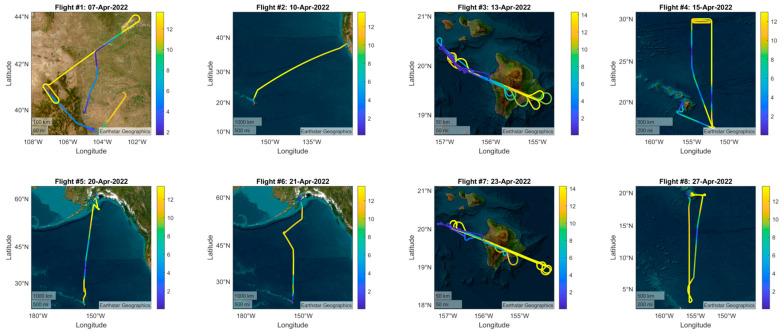
The eight flights conducted during TI3GER campaign, where MTHP was fully operational, acquiring data together with other atmospheric sensor and in situ data. The color bar represents the flight altitude. The color scale for the plots is the flight altitude in kilometers.

The TI3GER campaign facilitated the execution of a series of eight flights where MTHP was operated [26]. Throughout these flights, data were collected not only with MTHP but also with other several atmospheric sensors, as well as collocated in situ measurements using balloons. The comprehensive dataset from these flights can be accessed and explored in detail at the specified reference [23]. The campaign itself was conducted in April 2022, covering locations in multiple regions within the United States, including Boulder, Colorado; Hawaii; and Alaska. These diverse geographical areas were selected strategically to capture a broad range of atmospheric conditions and provide a comprehensive perspective on atmospheric dynamics. The eight flight paths followed during the TI3GER campaign are documented and depicted in Figure 9.

### 4.2. Preliminary Results

**Figure 10:**
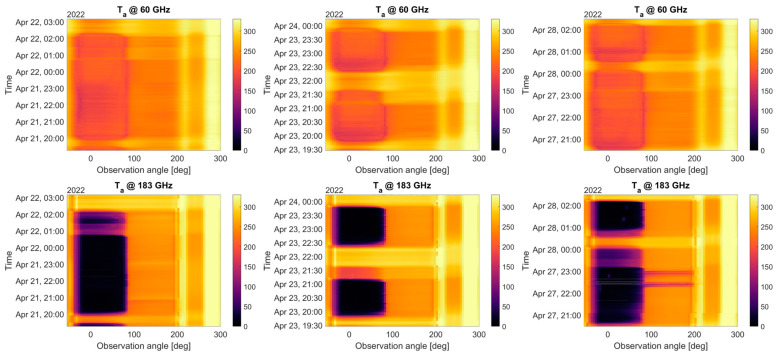
Detailed antenna temperature estimations (averaged for all frequency bins) for flights #6 (**left**) (Anchorage to Hawaii), #7 (**center**) (Hawaii island), and #8 (**right**) (Hawaii to Equator) evolution over time and as a function of the observation angle (zenith = 0°, nadir = 180°). The average airplane pitch is ~2.5°. Top row corresponds to the 60 GHz channel and bottom row to the 183 GHz channel.

To illustrate the preliminary results from the campaign, flights #6, #7, and #8 (see Figure 9) are selected for further analysis. Figure 10 shows the antenna temperature estimations after calibration for both 60 GHz and 183 GHz channels. To ease the visualization, all frequencies within each channel have been averaged together. The temperatures on the right-hand side of each plot correspond to the two internal loads: The warmed load, at a constant temperature of 333 K, and the ambient load, of which the temperature varies depending on the aircraft’s altitude. The antenna temperature also exhibits a drop in both channels for low observation angles (zenith-facing), and a sharp transition between the zenith-facing angles (0° to 85°) and the nadir-facing angles (95° to 180°), going from below 150 K to 220–250 K in both oxygen and water vapor channels. In this case, the water vapor channel (183 GHz) exhibits a lower temperature while facing the zenith when the plane is situated at altitudes higher than 10 km, which is not the case for oxygen (60 GHz).

The oxygen spectra at high altitudes have a spectral richness not comparable with the water vapor channel. For this reason, it is crucial to visualize the measured oxygen spectra at these altitudes. To validate the MTHP results, the measured antenna temperature is compared to a simulated profile generated using the Atmospheric Radiative Transfer Simulator (ARTS) [27]. To be as realistic as possible, the simulation uses three different atmospheric profiles depending on the flight. For flight #6, since it goes from Alaska to Hawaii, the 1986 COSPAR International Reference Atmosphere (CIRA86) is used, using 45° as a latitude setting and April as a reference month. For flight #7, the same atmosphere is used but is referenced at the Kona Island latitude of 15°. Finally, during flight #8, a radiosonde was also launched along with the flight. In this case, the radiosonde data are used as a reference temperature. The other input parameters are summarized in Table 3.

**Figure 11:**
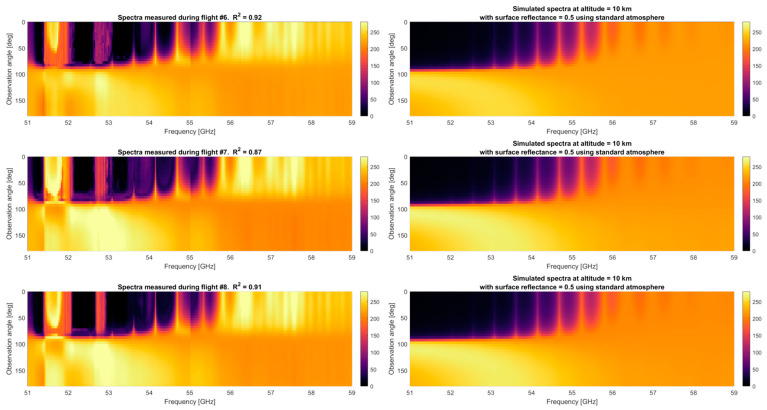
Detailed spectra measured during three flights compared to the ARTS simulated brightness temperature. Note that the 51.5 GHz to 52 GHz and the 52.5 GHz to 53 GHz regions are discarded due to RFI. Also, the region around 55 GHz is compromised due to the Nyquist filters not being sharp enough, folding outside the band inside the measurement.

The comparison between the measured spectrum and the simulated one is shown in Figure 11. Note that to visualize the spectra with respect to the visualization angle, the average of the entire flight whenever the airplane is at an altitude above 10 km is used (~2 h of data). As can be seen, the correlation between the simulated spectra and the retrieved profile is R^2^ = 0.92 for flight #6, R^2^ = 0.87 for flight #7, and R^2^ = 0.91 for flight #8. The correlation coefficient is computed after correcting bias and offset using a least-squares fit with robust regression. The first noticeable features of the measured spectra from MTHP are anomalies at 51.5 to 52 GHz and 52.5 to 53 GHz. We analyzed and concluded an anomalous receiver temperature in this area post-flight, which should not be used for retrieval purposes. Nonetheless, several areas show good agreement with the simulated profile in both the magnitude and spectral shape.

**Figure 12:**
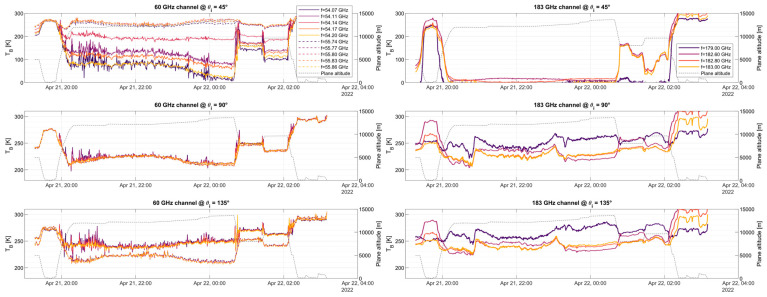
Measured Antenna Temperature for 10 selected channels (bin size is 31.25 MHz each, with $B_{N_{eq}}=$ 34.6 MHz) at the 60 GHz band receiver and for the 4 analog channels ($B_{N_{eq}}$ = 250 MHz each) of the 183 GHz band receiver. Flight displayed is #6, from Anchorage, AK, USA to Kona, HI, USA. Flight height is included in the right axis for reference.

**Figure 13:**
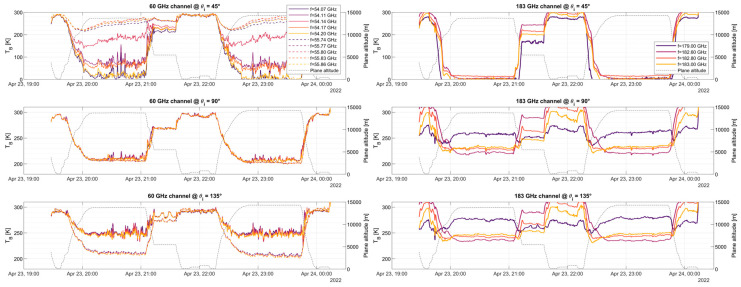
Same as Figure 12 but for flight #7, next to Kona, HI, USA.

**Figure 14:**
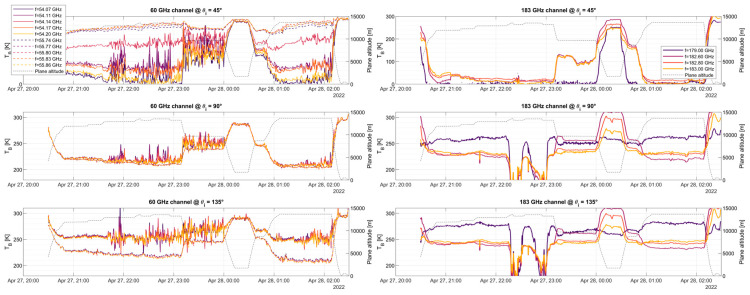
Same as Figure 12 but for flight #8, from Kona, HI, USA to the Equator, and back.

The zenith-facing part exhibits very good agreement with the simulation, showing the oxygen absorption band narrowing for higher altitudes at 53.07, 53.60, 54.14, 54.66, 55.30, and 55.80 GHz, higher frequencies exhibit a wider peak even at 10 km altitude. It is also worth noting a slight “glitch” or “jump” in the brightness temperature around 55 GHz. Note that the spectral area surrounding 55 GHz is where the two bands (upper and lower bands) used to sample the two 4 GHz portions of the spectra to produce an 8 GHz bandwidth meet. Due to the pre-sampling Nyquist filter not having a sharp cut-off and the signal an alias, this area shall not be used for science retrievals. The flights provided in Figure 10 and Figure 11 are shown as time series on a pseudo-multi-spectral look in Figure 12, Figure 13 and Figure 14. Each figure displays the time series of flights #6, #7, and #8 for the two receivers, at three selected angular positions (45° up-facing, 90°, front-facing, and 135°, which is 45° facing down, i.e., ±45° and forward). For the hyperspectral channel (60 GHz), high-resolution data are provided around two absorption lines, f = 54.14 and f = 55.80 GHz, with a total of five frequencies around each peak. For the water vapor channel, all four channels are displayed.

By analyzing the three figures, we can first see the absorption line antenna temperature when facing the +45° incident angle at f = 54.14 GHz, with an antenna temperature higher than its adjacent frequencies, f = 54.11 and f = 54.17 GHz. It can be also seen that the temperatures at the left- and right-hand sides of the peak are similar and within a few Kelvin (compare f = 54.07 and f = 54.20 GHz). For the higher peak (f = 55.80 GHz), the temperature of the peak is also larger than its adjacent frequencies, but with lower differences. It is worth mentioning that when the airplane is at low altitudes, all frequencies show the same temperature since the absorption line broadens near sea level. At front-view, the air cross-section measured by the antenna is maxima, hence all data measure the same value, namely, that of SAT. For the 135° (−45°) view, we can see that the lower frequency (solid line) measures a higher temperature as compared to the dashed line at the two selected absorption lines, which might be related to the fact that we are seeing the surface. It is also important to note that there is no noticeable difference between adjacent channels, as the absorption lines broaden while facing down.

Similar features can be identified in the 183 GHz channel. First, in the up-facing view for high altitudes, the measured temperature is near 2.73 K (cosmic radiation). Since there is little water vapor above 14 km, there is practically no signature of it at high altitudes. For down-facing measurements, it can be seen that the three adjacent measurements next to the absorption peak (182.6, 182.8, and 183 GHz) show a similar behavior, while the measurement located far from the peak (179 GHz) shows a different pattern. In this case, the three frequencies near the peak become highly attenuated by the presence of water vapor, hence, they measure the water vapor in view of the atmosphere. However, since the 179 GHz subchannel is mostly out of the water vapor absorption line, it does not get attenuated by its presence. Hence, this channel is potentially measuring the Earth’s surface emissivity, which can be used for calibration purposes.

### 4.3. Radio Frequency Interference

It is also important to note in flight #8 (Figure 14) the increased noise that is not present in other areas or flights in the lower frequency absorption line (~54 GHz), notably while facing upwards while the airplane is at a high altitude. Flight #8 was conducted from Kona to the Equator and back. Preliminary analysis of these results determined the potential presence of Radio Frequency Interference (RFI) coming from the coordinated inter-satellite link channel from the FCC (see 5.556A) [21]. The frequencies between 54.25 and 56.9 GHz are reserved for inter-satellite links between geostationary satellites.

## 5. Discussion

**Figure 15:**
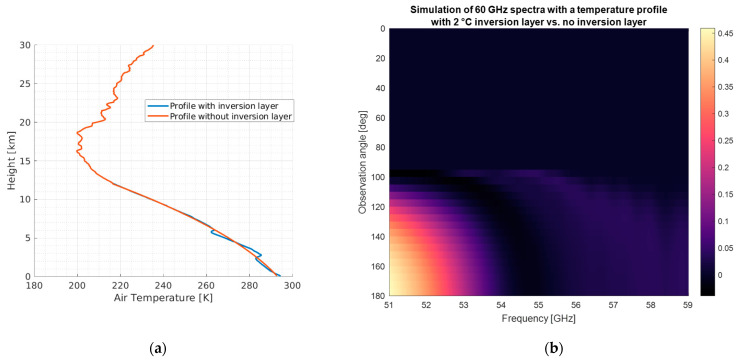
Simulation of a MTHP-like spectrogram with two profiles, one with an inversion layer and another one without the inversion layer. (**a**) The temperature profile used and (**b**) the difference between the profile with inversion layer and the profile without inversion layer.

Passive millimeter-wave sounding has been a longstanding approach to observing temperature and humidity profiles. However, state-of-the-art systems are bandwidth-limited and provide relatively coarse profiles that are not sufficient to address the challenging resolution requirements demanded by the PBL science community. Information content analyses have demonstrated the capabilities of hyperspectral approaches to increase the density of overlapping weighting functions thereby increasing retrieval resolution [28,29]. A similar effect to the increased overlapping weighting function can be achieved by viewing the same Earth target from multiple view angles. This high-density measurement together with the multangular information, which provides independent atmospheric information, might lead to a PBL estimation with a finer vertical resolution of around ~350 m, according to simulations using radiosondes. To support MTHP measurements being capable of eventually retrieving PBL information, we have computed a simulation of two spectra, one computed with an inversion layer and another one computed without an inversion layer using the frequency and angular resolution of MTHP. The simulation is provided in Figure 15. As can be seen, small differences in the bottom part of the atmosphere can be detected by the lowest part of the spectrum up to 0.45 K for the simulated case.

## 6. Conclusions

In conclusion, the manuscript discusses the development and deployment of MTHP for measuring vertical temperature and humidity profiles. The instrument design and components, including the dual-band receivers, are detailed. The calibration scheme, involving both internal calibration loads and static air temperature readings, is presented along with results from ground-based calibration tests. The manuscript highlights the successful execution of MTHP in the TI3GER campaign, providing preliminary results that show good agreement between measured and simulated profiles in various flight conditions. The measurements at the 60 GHz complex oxygen band are compared to ARTS-modeled brightness temperature measurements, showing a substantial agreement between both in all angular positions. The potential impact of RFI on the measurements is also presented, stressing the importance of reviewing and protecting radiometric bands used for atmospheric research. Overall, the MTHP presents a valuable tool for future atmospheric research projects, contributing to our understanding of the Planetary Boundary Layer and its role in the Earth system.

## Figures and Tables

**Table 1:** MTHP measured Y-factor and noise-equivalent temperature calculations.

Frequency	60 GHz		183 GHz			
	51–55 GHz	55–59 GHz	183 GHz	182.8 GHz	182.6 GHz	179 GHz
**Power measurement @ 293 K [dBm]**	−14.75	−16.30	−21.24	−20.99	−21.60	−23.68
**Power measurement @ 80 K** **[dBm]**	−16.04	−17.60	−21.98	−21.76	−22.37	−24.39
**Y-factor [dB]**	1.29	1.30	0.78	0.74	0.77	0.77
**Receiver** **Temperature [K]**	535.9	530.4	1066.6	1018.0	1018.0	1119.3

**Table 2:** NETD estimations for the MTHP receiver.

Channels	60 GHz		183 GHz			
	51–55 GHz	55–59 GHz	183 GHz	182.8 GHz	182.6 GHz	179 GHz
NETD [K]	3.63	3.60	0.89	0.83	0.83	0.86

**Table 3:** ARTS simulation parameters.

Parameter	Value
Altitude	10 km
View angles	0° to 180° (5° bins)
Frequencies	51 to 59 GHz in 31.25 MHz bins ($B_{N_{eq}}=34.6$ MHz)
Surface reflectivity	0.5
Atmospheric Profile	Flight dependant: #6: CIRA86 latitude 45°/April #7: CIRA86 Kona Island, latitude 15°/April #8: Radiosonde

## Data Availability

MTHP data are available at https://doi.org/10.26023/KH90-45VV-V905 free of charge for use.
